# A Compact Monitor for Ethylene and Other Plant-Produced Volatile Organic Compounds for NASA Space Missions

**DOI:** 10.3390/s23249713

**Published:** 2023-12-08

**Authors:** Vladimir Dobrokhotov, Alexander Larin, Elena Viugina, Adam Emberton, Andrey Livchak, Jay T. Cremer, Charles K. Gary

**Affiliations:** 1 Applied Physics Institute, Western Kentucky University, Bowling Green, KY 42101, USA; adam.emberton@wku.edu; 2 Halton Group, Scottsville, KY 42164, USA; alexander.larin@halton.com (A.L.); andrey.livchak@halton.com (A.L.); 3 Adelphi Technology LLC, Bowling Green, KY 42101, USA; eviugina@adelphitech.com (E.V.); ted@adelphitech.com (J.T.C.J.); cgary@adelphitech.com (C.K.G.)

**Keywords:** chemical sensors, gas chromatography, ethylene, volatile organic compounds

## Abstract

In this work, we discuss the development of a compact analytical instrument for monitoring ethylene in compact greenhouses utilized by NASA to grow fresh vegetables in space. Traditionally, ethylene measurements are conducted by GC-MS systems. However, in space, they are not applicable due to their bulky size, heavy weight, special carrier gas requirement and high maintenance. Our group developed a compact and robust battery-powered ethylene monitor based on the principles of analytical gas chromatography. The device utilizes purified ambient air as a carrier gas and a metal oxide sensor as a GC detector. Implementation of a CarboWax 20 M packed column from Restek together with a Tenax TA pre-concentrator allowed us to achieve a 20 ppb limit of detection for ethylene. Full automation of measurements and reporting of concentrations was accomplished via the implementation of a Raspberry Pi 4 computer and a 7″ 720P LED capacitive touchscreen utilized for data output. Based on a feasibility study, a fully automated, industrial-grade ethylene monitoring and removal system for greenhouses was developed.

## 1. Introduction

Human missions in space, from the International Space Station to potential human exploration of the moon, Mars and beyond, will require advanced systems to maintain an environment supporting human life. Smart greenhouses are, therefore, necessary for a fresh food supply in long-term space missions [1]. A key function is to reproduce the Earth’s natural ecosystem, aiming at the “closure” of the air, water and food cycles in a so-called closed ecological life support system (CELSS) [2]. The main demonstrated advantages of CELSSs over more classical physicochemical systems are both psychological and logistic. The “Earth-like” habitat and the production of fresh food are quite attractive features for humans in very confined and harsh environments. Plant health can be monitored in closed ground or space habitats by means of a few critical parameters: light conditions, CO_2_, humidity, volatile organic compounds (VOCs) and ethylene emissions [3]. Ethylene is the simplest alkene molecule (molecular formula: C_2_H_4_) that has four hydrogen atoms bound to a pair of carbon atoms [4]. The ethylene molecule has a comparably small kinetic diameter of 4.163 Å, and bond lengths of carbon to hydrogen and carbon to carbon of 1.096 and 1.335 Å, respectively [5,6]. It has a lighter specific weight (1.225 kg/m^3^) with respect to air (1.178 kg/m^3^) at 15 °C [7]. Ethylene is freely diffused in ambient air with an approximate concentration of 2 parts per billion (ppb). Ethylene plays a significant role in engineering, environmental, health, agriculture and food science due to its physical and chemical functionality. Detection of ethylene in real time is especially important in agriculture [6]. Ethylene is a well-known natural phytohormone.

Plants can produce ethylene through natural metabolic processes, and this ethylene can accumulate in closed environments (such as closed plant growth chambers) and have undesirable effects on the plants. In addition to plants, some micro-organisms, including fungi and bacteria, can synthesize ethylene themselves. In these conditions, it is of great importance to elucidate the role played by ethylene released by each agent during the pathogen–host interaction. *Botrytis cinerea* is one example of an important worldwide pathogen, which attacks more than 200 plant species and causes extensive losses to many field-grown and greenhouse crops.

Ethylene significantly affects and regulates plant and fruit development stages such as root growth [8], seed germination [9], fruit ripening [10,11,12,13], flower senescence [14,15] and oxidative stress levels [16]. Ethylene effects on plants can also include reduced growth, impaired pollen development and/or fertilization, leaf epinasty and flower abortion [17]. According to the latest research, some fruits (apples, bananas, tomatoes, etc.) ripen with an ethylene concentration between 0.1 and 1 parts per million (ppm) [13], and flowers usually start to blossom under 0.1 ppm [14,15]. Being hormonal in nature, ethylene can affect plants at very low concentrations, with levels as low as 25 ppb being reported to have subtle effects on some plants.

Control of ethylene levels is an important part of the next generation of greenhouses, food processing facilities and storage infrastructure that would help to minimize the waste of food products. Some sophisticated plant growth chambers for NASA applications include ethylene removal systems, such as KMnO_4_-coated pellets, but this is a consumable material and adds resistance to air circulation in the chamber. Real-time ethylene monitoring allows for a more judicious use of ethylene removal for the control of plant growth, saving consumables.

State-of-the-art technologies for ethylene detection include analytical gas chromatography (GC), Fourier transform infrared technology (FTIR), photonic-crystal-fiber-enhanced Raman spectroscopy, surface acoustic wave (SAW) analyzers, photoacoustic sensors, printable colorimetric sensor arrays and a wide range of nanostructured chemoresistive gas sensors and metal-oxide-based sensors [18,19,20,21,22,23,24,25,26,27,28,29,30]. Out of all the detection methods, gas chromatography, electrochemical cells and optical sensors were found to be the most promising for their high sensitivity, selectivity and stability. Optical-laser-based methods are very sensitive and selective with a short time of response, but very costly. Electrochemical cells have multiple advantages including a high selectivity and accuracy, a relatively short response time, and a high sensitivity at a low parts per billion level. However, the low operational lifetime (3–6 months) of electrochemical cells is a major disadvantage of this method. Real-time field monitoring using a conventional GC with standard flame ionization detectors (FIDs) or photo ionization detectors (PIDs) also has several fundamental barriers and limitations due to their bulky size, heavy weight, special carrier gas requirement and high maintenance. The special carrier gas requirement (bulky gas tanks need to be attached to the instrument for operation) is, probably, the major drawback of conventional chromatography, limiting its portability.

The needs for monitoring in space require a paradigm shift in gas analyzer design. The device should have the capabilities of a comprehensive analytical instrument and at the same time should be compact, portable and robust. On-site analysis of volatile organic compounds (VOCs) with compact GC systems is a rapidly developing technology. Compact gas chromatographs are constantly improving thanks to novel materials, process developments, miniaturization, and optimization of power consumption. The GC method for the detection of ethylene in plants and fruits can be broadly utilized in the agricultural industry due to its high sensitivity (<15 ppb), selectivity, simultaneous analysis of multiple analytes, long lifetime, reliability (1–2% accuracy), portability (5–10 lb.), and fast results within a few minutes.

The latest studies demonstrated promising results for ethylene pre-concentration, allowing GCs to achieve the limit of detection at sub-ppb levels for shelf-life prediction of climacteric fruits [31]. At the same time, the portable GC method is highly selective thanks to the separation of mixtures by a chromatography column. Novel compact GCs are capable of detecting ethylene under a variety of environmental conditions (temperature, humidity and dust) due to the preconditioning of the carrier gas [32].

Despite the multiple advantages, state-of-the-art compact GCs require a highly qualified operator for sample collection and data analysis. The development of user-friendly, remotely controlled GCs with multiple sample collection points would minimize the operator involvement. Also, it would allow a single operator to collect and analyze gas samples from multiple locations. Fully automated GC systems can be controlled by artificial intelligence (AI) and the analysis of gas samples can be performed either on demand or on schedule. The performance characteristics of a compact GC can be improved drastically by implementing full process automation by using enhanced signal processing and data analytics capabilities, by utilizing cloud resources, and by integrating self-diagnostics and maintenance services into the system infrastructure.

In this work, we present a compact battery-powered analytical instrument for monitoring ultra-low concentrations of ethylene in complex backgrounds. The device architecture and operation are based on the principles of analytical gas chromatography. The key advantages of our monitoring technology are the utilization of scrubbed ambient air as a carrier gas and the utilization of a metal oxide (MOX) sensor as a GC detector [33,34,35,36,37,38]. By implementing a Tenax-based pre-concentrator, we were able to achieve a 15 ppb limit of detection (LOD) for ethylene.

Based on the feasibility prototype, we developed an industrial-grade system for ethylene monitoring and removal from multiple greenhouses on a spacecraft. Our greenhouse monitoring system is a compact unit, which is robust, has a high environmental tolerance and is suitable for mass production. In this system, a novel industrial-grade control platform (DISTECH, ECY303-M3, Brossard, QC, Canada) for compact gas analyzers was utilized to provide a universal control solution for GC operation, web interface, data integration, process automation and multi-channel sampling.

## 2. Materials and Methods

The mechanism of the MOX sensor’s response to volatile chemicals is based on a catalytic reaction with oxygen species at the surface [39,40,41,42,43,44,45,46,47,48]. At temperatures between 150 and 400 °C, oxygen is adsorbed and ionized at the surface’s active sites by trapping electrons from the bulk, with the overall effect of increasing the resistance of the sensor. The occurrence of the analyte of interest in the atmosphere, which reacts with the adsorbed oxygen, determines a change in the sensor resistance, the magnitude of which is correlated to the concentration of the analyte.

The use of metal oxide sensors as GC detectors has the advantage of enabling the use of scrubbed ambient air as the carrier gas for the GC column. This is a great advantage for a portable GC, because the special carrier gas requirement (bulky gas tanks need to be attached to the instrument for operation) is, probably, the major drawback of conventional chromatography, limiting its portability. A highly sensitive non-specific MOX sensor, a TGS 822 fabricated by FIGARO (Rolling Meadows, IL, USA), was utilized as a GC detector for this study.

Another advantage of metal oxide sensors is their effective lifespan of approximately 10 years, which is significantly longer compared to PID detectors and electrochemical cells. In the compact GC, the carrier gas is purified dry air that undergoes moisture removal with a moisture trap and background VOC removal with a carbon-based filter (air scrubber). Under these conditions, the lifespan of MOX detectors can exceed 10 years.

**Figure 1:**
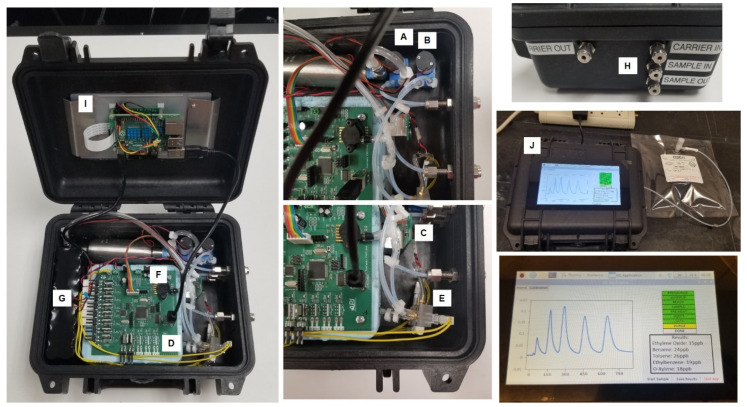
Internal architecture of the VOC analyzer. (**A**) Carrier gas generation pump, (**B**) sample pump, (**C**) detector module, (**D**) GC column oven assembly, (**E**) injector module with pre-concentrator and cooling fan, (**F**) microprocessor control board, (**G**) internal battery, (**H**) sample inlet, (**I**) Raspberry Pi 4 computer, (**J**) 7″ 720P LED touchscreen.

**Figure 2:**
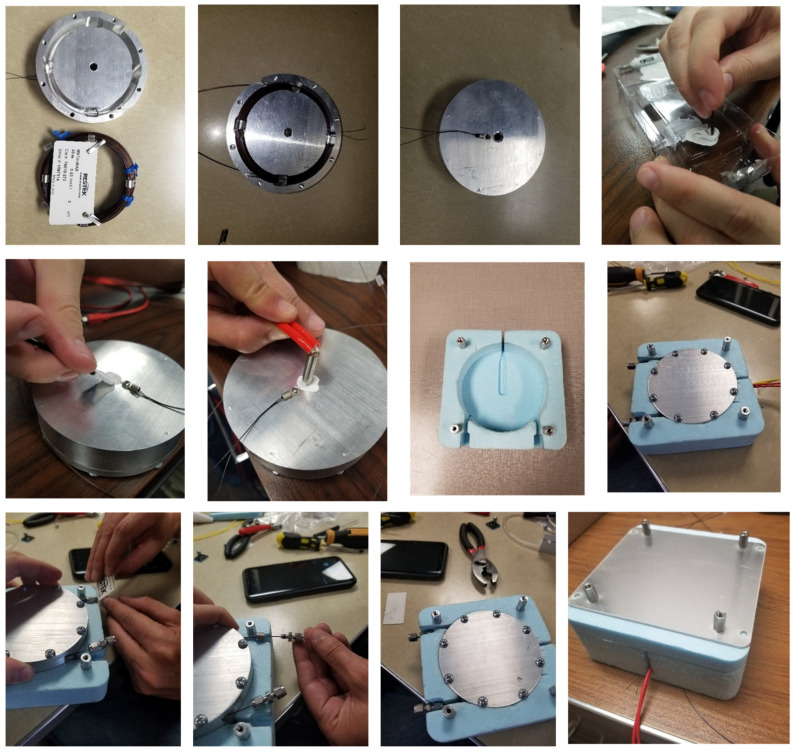
Assembly of the GC column module: Column is first placed in a metallic oven. Next, a resistive heater cartridge is placed inside the oven via the center opening and secured with thermal adhesive. Then, the oven is placed inside the polystyrene foam thermostat.

The internal architecture of the analyzer is shown in Figure 1. The assembly of the column module is shown in Figure 2. The column is first placed in a metallic oven. After that, a resistive heater cartridge is placed inside the oven though the opening at the center and secured with thermal adhesive. After that, the oven is placed inside the polystyrene foam thermostat. The column module in this form can be easily connected to the gas delivery system of the analyzer as well as to the power supply for the heater.

Three columns were extensively tested for ethylene extraction and quantification (see Table 1). The goal of testing was to identify a column satisfying the following criteria:

- Capable of extracting ethylene from the most complex background mix.
- Capable of separating ethylene from the light compounds, especially plant-produced compounds, such as methane, hydrogen sulfide and ammonia.
- Capable of separating and quantifying the greatest number of plant-produced VOCs.

It was found that the CarboWax packed column from Restek (Bellefonte, PA, USA) satisfies all three criteria. The following operational conditions were implemented: a flow rate of 14 sccm (standard cubic centimeters per minute) of clean dry air and a constant column temperature of 74 °C for CarboWax. The operational temperature of the detector was kept at 250 °C.

To reduce the limit of detection, a Tenax TA pre-concentrator was utilized. Tenax TA is a porous polymer based on 2,6-diphenyl-p-phenylene oxide, and is widely used as an adsorbent in both air collection and purge trap applications. Its unique structure provides alternate and desirable adsorption/desorption characteristics compared to other porous polymers. It holds up well for multiple desorptions without much bleed, which is especially important for highly sensitive detectors.

**Figure 3:**
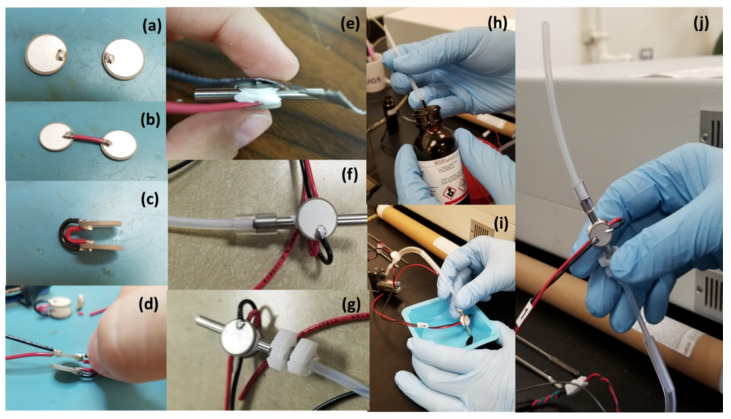
Pre-concentrator assembly: (**a**–**c**) Soldering of inside and outside pads of two positive temperature coefficient (PTC) resistors with red and black wires, respectively. (**d**) Soldering 12″ segment of red and black wire to the inside and outside pads of PTC resistors, respectively. (**e**) Thermal epoxy applied to the inner sides of PTC heaters and to the middle of 1/8″ stainless steel tubing. (**f**,**g**) Assembly of pre-concentrator using silicone tubing and Teflon tubing. (**h**,**i**) Filling the pre-concentrator with sorbent material. (**j**) Assembly complete.

The pre-concentrator is a short segment of a metallic tube, filled with 40 milligrams of Tenax and equipped with two positive temperature coefficient (PTC) elements, allowing for a quick increase in the sorbent temperature. The pre-concentrator assembly is shown in Figure 3. Analytes are trapped by the Tenax sorbent at room temperature, after which the molecules thermally desorb at 100 °C and are redirected through the GC column. 

**Figure 4:**
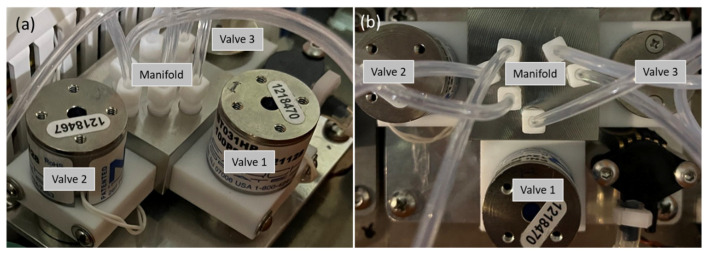
The side (**a**) and the top (**b**) view of the injector module: three-way solenoid valves with the stainless steel manifold.

A system of three-way solenoid valves manufactured by NResearch Inc. (West Caldwell, NJ, USA) and a stainless steel manifold constitute the injector module (Figure 4). The injector is used to redirect the air flow during the GC operating cycle. This solution provides several advantages such as a low cost, a compact size, easy/low maintenance, and high reliability. In this study, the injector was operated at room temperature, since all the compounds of interest were volatile. The injector can operate at elevated temperatures for analysis of heavy compounds and semi-volatiles. 

**Figure 5:**
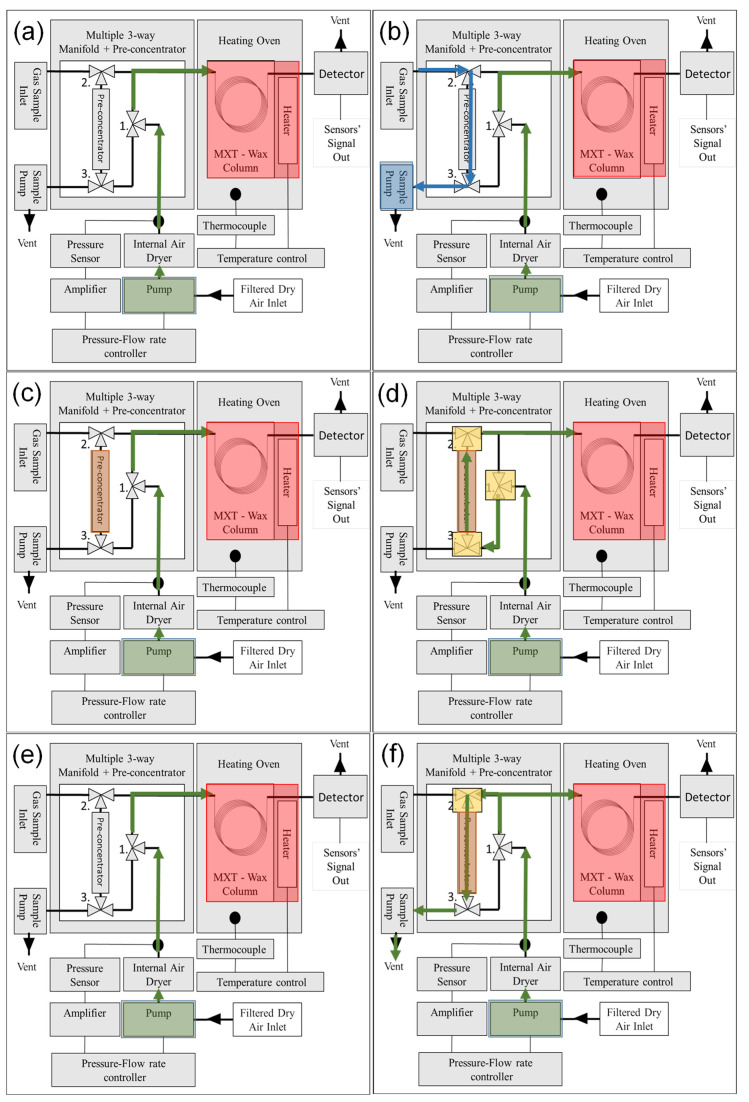
Operating cycle of the analyzer: (**a**) turning on, (**b**) sampling, (**c**) desorption, (**d**) injection, (**e**) analysis, (**f**) purging.

The operating cycle of the analyzer is shown in Figure 5. When the power switch of the device is turned on, the main pump activates a constant 10 mL/min flow of carrier gas (scrubbed air) through the column (Figure 5a). The carrier gas first flows through the cylinder with desiccant and then through the cylinder with scrubber; then, it flows through valve 1 and a metallic T-element to the column and from the column to the detector module. The carrier gas is flowing through the system at all times when the device is on. Once the column temperature stabilizes, the analyzer is ready for sampling. When the sample pump is activated, the ambient air containing a mixture of gases passes at 10 mL/sec through a pre-concentrator, which collects the molecules of interest (Figure 5b). In this phase of the cycle, both valves 2 and 3 are open and the pre-concentrator is at room temperature, so it efficiently absorbs the chemicals. The typical sampling time for our device is 10 s. After 10 s, the system continues to the next step automatically. Once the sampling is complete, valves 2 and 3 close and the pre-concentrator heats up to 100 °C to desorb the molecules (Figure 5c). After 30 s, the system continues to the next step automatically. In the injection part of the cycle, the carrier gas is redirected by valve 1 through the pre-concentrator (Figure 5d). Valves 2 and 3 are open and the pre-concentrator remains at a high temperature during this step, so that the desorbed gas sample is injected into the column. The injection volume is 0.4 mL. After the injection is complete, the system switches to analysis mode. In the analysis part of the cycle (Figure 5e), valve 1 redirects the carrier gas directly through the column, bypassing the pre-concentrator. The analyte mixture is separated by the column.

The analytes exit the column and hit the detector. The detector signal is measured and recorded. The time of this phase is determined by the retention time of the heaviest analyte in the mix. After that, the system continues to the next step automatically. After the analysis is complete, the device purges the pre-concentrator of the remaining particles (Figure 5f). Valve 2 opens to branch the flow from the main pump through the pre-concentrator and out of the sample pump. In this phase, the pre-concentrator remains hot to desorb the remaining particles. The sample pump is not powered; the pressure of the gas allows the gas to escape thorough the pump without assistance. This step concludes the entire operational cycle of the GC, and the device is ready for the next scan.

## 3. Results

The CarboWax Carbon Molecular Sieve (CMS) is a novel material based on through-pore structures. Specially designed pores taper from macro- to meso- to micropores, resulting in materials with excellent thermodynamic properties for both adsorption and desorption processes. The CarboWax column from Restek provides the best performance for separation of a wide range of plant-produced VOCs.

**Figure 6:**
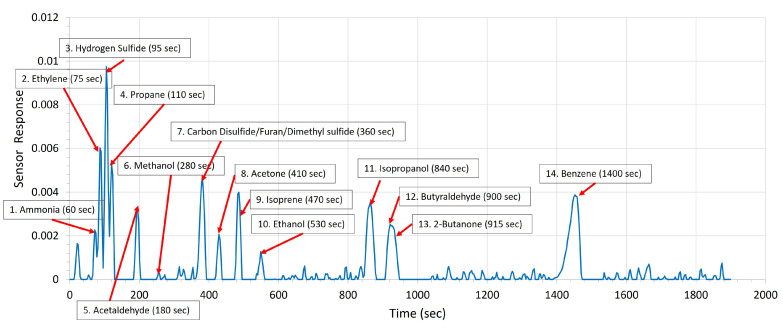
Separation of plant-produced VOCs by the CarboWAX column at 74 °C.

Traditionally, the time separation of light compounds has been problematic for non-MS gas chromatographs utilizing compact detectors. Light compounds tend to coelute and come out as a single peak. CarboWax was found to be an excellent solution for separating mixtures of small molecules, with a clear ethylene peak (Figure 6).

The reported result was obtained under the constant column temperature of 74 °C. The GC is equipped with programable temperature control. Temperature ramping can be applied if needed for better separation of chemicals or for purging the remaining heavy components at the end of each GC cycle.

**Figure 7:**
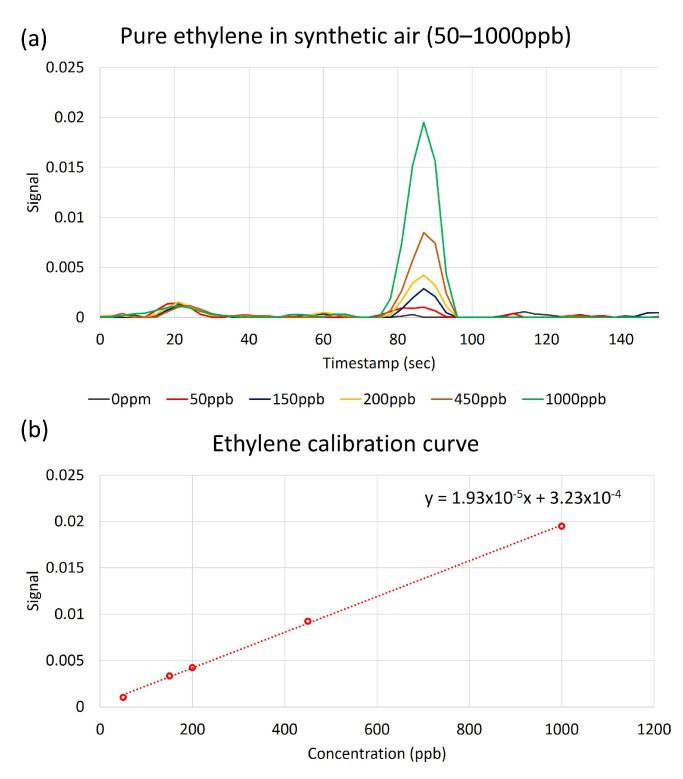
(**a**) Chromatograms of pure ethylene diluted in synthetic air. (**b**) Calibration curve: ethylene peak vs. concentration.

**Figure 8:**
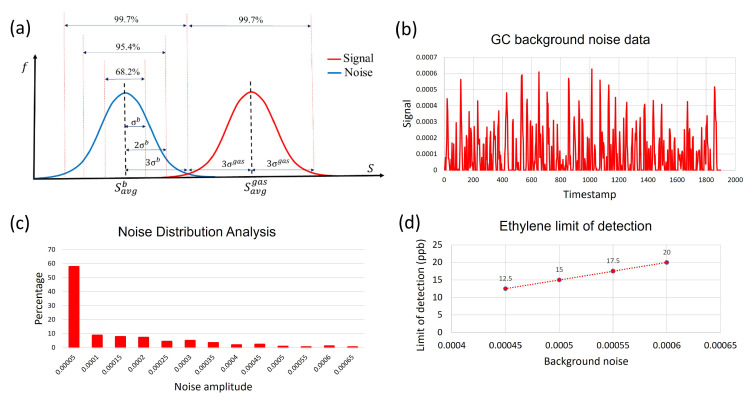
(**a**) LOD concept. Concentration corresponding to lowest detector response (red curve) that can be distinguished from detector noise (blue curve) is the LOD. (**b**) A segment of the background noise data. (**c**) Distribution of various noise amplitudes. A total of 99.7% of the noise spikes have amplitudes below 0.0006. (**d**) Illustration of the potential sensitivity increase by reducing the noise.

In our experiments, device calibration was conducted by collecting chromatograms of pure ethylene diluted in synthetic air, shown in Figure 7. The minimum limit of detection (LOD) for ethylene with a confidence level of 99.7% was determined by analyzing the drift in the sensor’s baseline and sensor’s response in a 2-week period. In this analysis, the normal distribution function was used to find the average value and the standard deviation of the baseline over time and variations in the sensors’ response over time for multiple identical exposures. A low detection limit is defined as the minimum measurable signal exceeding the threshold. The threshold is equal to the mean value of noise plus three standard deviations of the noise. The distance of three standard deviations from the mean value corresponds to 99.7% of the area under the normal distribution curve, Figure 8a. 

This choice of parameters assures that the signal can be distinguished from noise with 99.7% probability. A segment of the background noise data and the distribution of various noise amplitudes are shown in Figure 8b,c, respectively. Based on continuous monitoring over a 2-week period, 99.7% of the noise spikes have amplitudes below 0.0006. All the points above this threshold can be considered as a signal with 99.7% probability or higher. Following this formalism, the LOD for ethylene in our experiments was determined as 20 ppb. By applying various noise reduction techniques, the ethylene detection limit can be further reduced. Figure 8d illustrates the dependence between the limit of detection and the background noise level.

**Figure 9:**
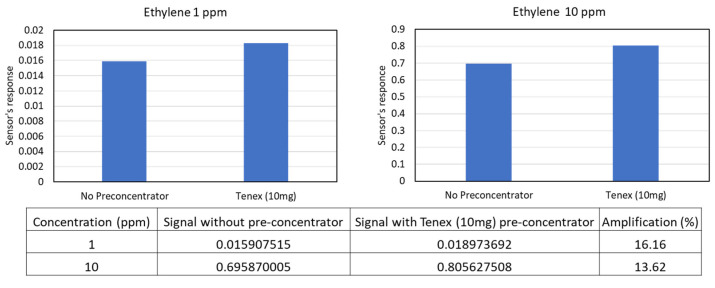
Analysis of signal amplification by the Tenax TA pre-concentrator.

The efficiency of using Tenax TA for ethylene pre-concentration was studied quantitatively. The detector response to direct sampling without a pre-concentrator was compared against the sampling with the added 10 mg Tenex pre-concentrator (Figure 9). Experiments were conducted at 1 and 10 ppm concentrations of ethylene. For 1 ppm, the observed signal amplification by pre-concentration was 16% and for 10 ppm, it was close to 14%. Hence, pre-concentration of ethylene with Tenax TA was relatively low compared to most VOCs. However, even this relatively low signal amplification was found to be useful for LOD reduction. In addition, for some of the VOCs of interest, the Tenax pre-concentrator provides up to a 10-fold signal amplification.

The GC shown in Figure 1 is fully automated so that the ethylene concentration can be measured without operator involvement. The firmware extracts the peak of interest from the chromatogram and calculates the corresponding concentrations using the calibration curve. A Raspberry Pi 4 computer (Raspberry Pi Foundation, Cambridge, UK) and a 7″ 720P LED capacitive touchscreen were utilized for data output. Once the cycle is complete, it reports the concentration of any detected ethylene in the sample. A push button user interface outputs data as the numerical ethylene concentration after each scan. Raw signal vs. time data can also be reported and/or stored.

## 4. Discussion

The ability to extract ethylene from multicomponent mixtures of almost any complexity with a compact device presents an opportunity for developing a system for ethylene monitoring and removal from multiple greenhouses on a spacecraft.

**Figure 10:**
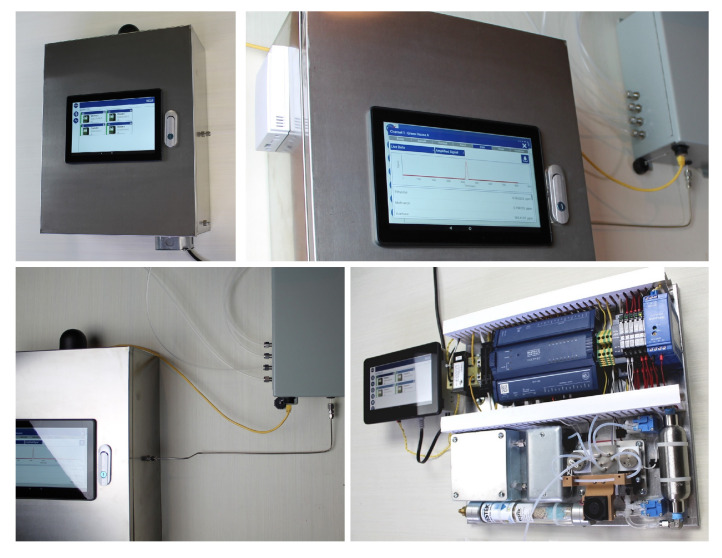
Industrial-grade compact Adelphi ethylene/VOC monitor based on a DISTECH controller.

The Adelphi greenhouse monitoring system is a compact unit in a metallic enclosure (Figure 10), which is robust, has high environmental tolerance and is suitable for mass production. In this system, a novel, industrial-grade control platform (DISTECH, ECY303-M3) for compact gas chromatograph analyzers was utilized to provide a universal control solution for GC operation, web interface, data integration, process automation and multi-channel sampling. The industrial-grade secure DISTECH controller naturally replaced the Raspberry Pi computer, which was utilized in the original prototype (Figure 1) and operates as the open-source ecosystem.

**Figure 11:**
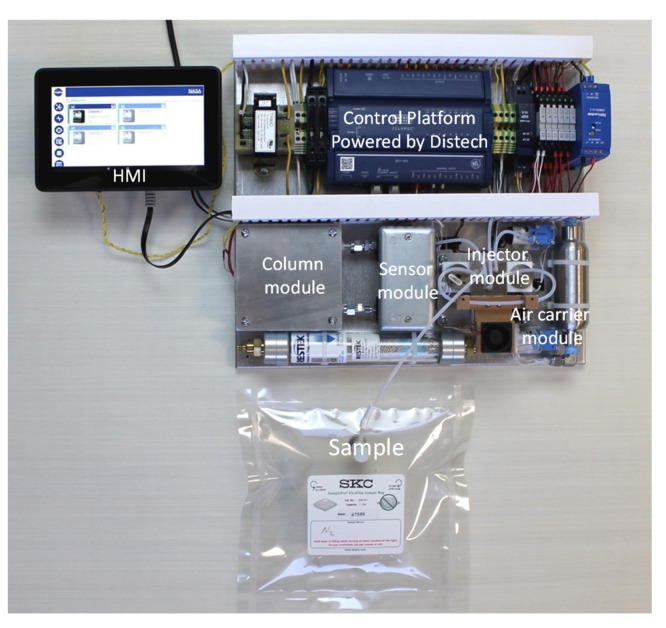
Structural optimization of GC components.

The structural optimization of the analyzer was achieved thanks to the implementation of a user-friendly modular design, shown in Figure 11. This design allows an operator to quickly interchange parts to achieve the desirable functionality or to conduct a quick on-sight repair. The internal architecture of the onboard gas analyzer is based on a system of quickly replicable blocks or modules, e.g., a pre-concentrator module, an injector module, a column module, a detector module and an air carrier module. The detector module, the injector module and the pre-concentrator module can easily be replaced by using two threaded Swagelok connectors to the solenoid valves. A module swap can be performed by a trained technician in under 5 min.

Switching between the column modules requires changes to the data processing software. Adelphi developed an interface which is equally convenient for users and developers. The “Application Library” is a standard preprogrammed database for the detection of a variety of chemicals. The database is uploaded to the device during the manufacturing process and then can be updated locally as a file or remotely as automatic updates. After the column module swap, the “Application Library” automatically configures the column temperature, flow rate, list of detectable chemicals, detector type and its activation voltage, retention time for each chemical and calibration curve (signal vs. concentration) for each chemical. An operator can easily change the column module, choose the new column in the software menu and begin monitoring.

Simultaneous monitoring of several greenhouses implies chemical data collection from multiple monitoring points. The most efficient solution for a such distributed monitoring would be through the utilization of a tube bundle system with a single device rather than using multiple devices, especially considering the volume and mass limitations for equipment on a spacecraft.

A tube bundle system (TBS) is a mechanical system for continuously drawing gas samples through tubes from multiple monitoring points. The gas samples are drawn via a sampling pump to the GC and the results of the gas analyses are displayed and recorded for further processing. The TBS is a well-developed technology and has been used in coal mines around the world for more than 50 years. Most longwall coal mines in Australia deploy a TBS, usually with 30 to 40 monitoring points as a part of their atmospheric monitoring [49].

**Figure 12:**
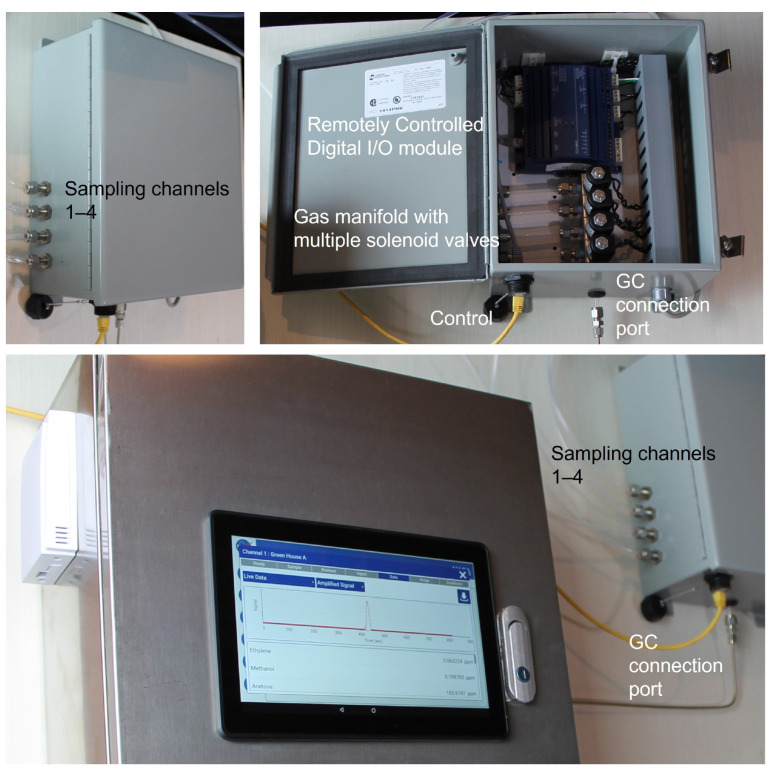
Multichannel gas sampling system for monitoring multiple greenhouses.

As the gas chromatograph, the Adelphi multichannel sampling system is also operated by a DISTECH controller (Figure 12). The controller supports communication with up to four remote ECx-Light modules, which could be installed at distance of one hundred meters from the main controller. Each module enables sampling from four independent sampling units/compartments. The current design maximum amount for sampling units is equal to 16 (4 × 4).

**Figure 13:**
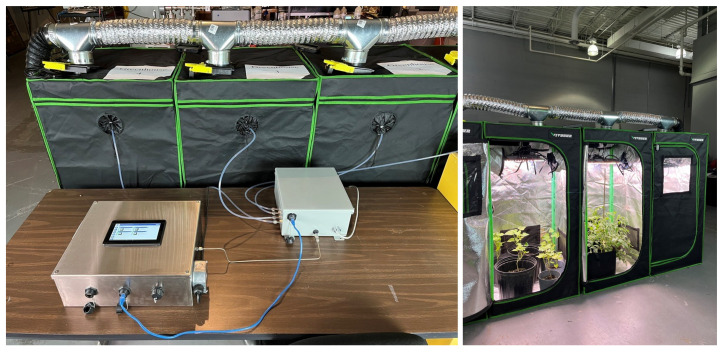
Monitoring of four compact greenhouses with a single chromatograph equipped with a multichannel gas sampling system.

Figure 13 shows the fully automated system for the monitoring and removal of ethylene and other plant-produced VOCs developed by Adelphi for NASA. The system is equipped with a distributed four-channel sampling system allowing the sequential monitoring of four greenhouses. Four VIVOSUN compact greenhouses were used to grow three types of vegetables: bell peppers (greenhouse #1, monitoring channel #1), sweet potatoes (greenhouse #2, monitoring channel #2) and tomatoes (greenhouse #3, monitoring channel #3). The fourth greenhouse was left empty for baseline control (monitoring channel #4).

**Figure 14:**
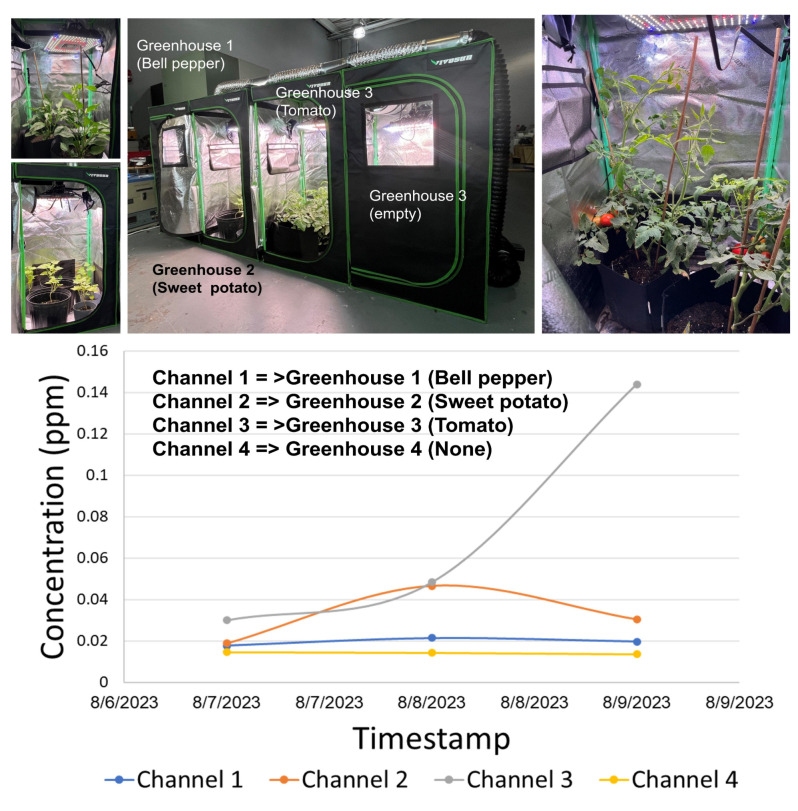
Ethylene accumulation in the greenhouses without venting for three consecutive days.

An example of ethylene monitoring in the greenhouses that were left without venting for three consecutive days is shown in Figure 14. The most significant increase was observed in greenhouse #3 (tomato), followed by greenhouse #2 (sweet potato) and greenhouse #1 (bell peppers). Tomatoes were monitored during the ripening period, so the intense ethylene release was expected. No ethylene was detected in the empty greenhouse #4. In this experiment, the greenhouses were not sealed and some natural ethylene leakage in the external medium was inevitable. In the case of the sealed NASA growth chambers, ethylene accumulation is expected to produce higher concentrations over a shorter time period.

The ethylene extraction and removal system was installed above the greenhouses (Figure 14). The controller activates the ventilation system of the greenhouse when the ethylene concentration reaches a certain threshold. On a spacecraft, ethylene removal systems utilize KMnO_4_-coated pellets, which is a consumable material and adds resistance to air circulation in the chamber. A real-time ethylene monitoring system controlling the ventilation would allow for a more judicious use of ethylene removal for controlling plant growth and saving consumables.

## 5. Conclusions

A compact, fully automated VOC monitor was developed. It is based on the principles of analytical gas chromatography, utilizes purified ambient air as a carrier gas and a MOX sensor as a GC detector. Use of a CarboWax column from Restek together with a Tenax TA pre-concentrator allowed us to achieve a 20 ppb limit of detection for ethylene. At the same time, the capabilities of the compact VOC analyzer go beyond the detection and quantification of a single compound. The modular design, allowing a quick swap of columns, makes our system capable of monitoring multiple plant-produced VOCs. VOCs are released through a variety of plant processes and vary greatly in chemistry and quantity though a plant’s life cycle. These compounds include numerous biogenic species, e.g., alcohols, isoprene, monoterpines, acids, carbonyls, alkanes, and alkenes [50,51,52]. In a closed environment, VOCs may create a toxic environment for either humans or other plants. Human responses to biogenic compounds may include acute toxicity, chronic toxicity, and allergenic effects. Chronic exposure to low concentrations of biogenic compounds, which might be common during extended space habitation missions, is largely unstudied and of particular interest.

## Figures and Tables

**Table 1:** Columns tested for ethylene extraction and quantification.

Column Name	Type	Coating Material	OD	ID	Column Length (m)	Mesh
CorboWax	packed/micropacked	CarboBlack B (S12) (Fisher Scientific, Hampton, NH, USA)	1/8 in	2.1 mm	2 m	80/120
CarboXen	packed/micropacked	Carboxen (Sigma-Aldrich, St. Louis, MO, USA)	1/8 in	2.1 mm	4.25 ft	60/80
MXT-BAC1	capillary	MXT™-BAC1 (Fisher Scientific, Hampton, NH, USA)	0.83 mm	0.53 mm	30 m	-

## Data Availability

Data are contained within the article.
